# Stimulating Old Fruit Trees

**Published:** 1886-08

**Authors:** 


### *STIMULATING OLD FRUIT TREES.*

When apple and other fruits trees lose vigor from age, or from neglect or want of fertilizing, there are different modes for restoring them to vigor, which may be used to the best advantage during autumn. One of the easiest of application is to spread a coat of barn manure broadcast for twenty-five or thirty feet distance from the trunk of the tree on each side. This treatment will frequently be sufficient of itself, in addition to pruning out any dead branches, or thining in the head from the outside, or both. Cultivating the surface while the tree is dormant is an additional aid. For smaller trees which are required to stand in grass, or where they cannot be cultivated, a strong impetus may be given by cutting radiating trenches from within a few feet of the tree a rod or so in length on all sides, and filling these with compost. The large roots, which lie on each side of these trenches, will send their side roots into the compost, and the trees will be pushed rapidly in growth. The trenches may be from one to two feet in depth, and the width that of the blade of the spade. Another mode, instead of cutting these radiating trenches, is to cut a circular ditch around the tree at some distance from the trunk and to fill this with compost. Several roots will soon send fibres into the compost, while the pruning which the roots receive will promote fruitfulness. Some judgement must be used to cut the ditch at the right distance ; if too near it will temporarily check the growth too severely, before new roots are formed ; and if too far away, the labor will be too great, and result less striking. In most cases, however, and with orchards of some size, cultivating and top-dressing will be sufficient. All cutting of the roots or branches mnst be done while the trees are not growing.—*Country Gentleman.*

